# Immunopathogenesis of Craniotomy Infection and Niche-Specific Immune Responses to Biofilm

**DOI:** 10.3389/fimmu.2021.625467

**Published:** 2021-02-23

**Authors:** Sharon DB de Morais, Gunjan Kak, Joseph P. Menousek, Tammy Kielian

**Affiliations:** ^1^ Department of Pathology and Microbiology, University of Nebraska Medical Center, Omaha, NE, United States; ^2^ Department of Neurosurgery, University of Nebraska Medical Center, Omaha, NE, United States

**Keywords:** biofilm, *Staphylococcus aureus*, craniotomy, myeloid-derived suppressor cell, neutrophil, macrophage, microglia

## Abstract

Bacterial infections in the central nervous system (CNS) can be life threatening and often impair neurological function. Biofilm infection is a complication following craniotomy, a neurosurgical procedure that involves the removal and replacement of a skull fragment (bone flap) to access the brain for surgical intervention. The incidence of infection following craniotomy ranges from 1% to 3% with approximately half caused by *Staphylococcus aureus* (*S. aureus*). These infections present a significant therapeutic challenge due to the antibiotic tolerance of biofilm and unique immune properties of the CNS. Previous studies have revealed a critical role for innate immune responses during *S. aureus* craniotomy infection. Experiments using knockout mouse models have highlighted the importance of the pattern recognition receptor Toll-like receptor 2 (TLR2) and its adaptor protein MyD88 for preventing *S. aureus* outgrowth during craniotomy biofilm infection. However, neither molecule affected bacterial burden in a mouse model of *S. aureus* brain abscess highlighting the distinctions between immune regulation of biofilm vs. planktonic infection in the CNS. Furthermore, the immune responses elicited during *S. aureus* craniotomy infection are distinct from biofilm infection in the periphery, emphasizing the critical role for niche-specific factors in dictating *S. aureus* biofilm-leukocyte crosstalk. In this review, we discuss the current knowledge concerning innate immunity to *S. aureus* craniotomy biofilm infection, compare this to *S. aureus* biofilm infection in the periphery, and discuss the importance of anatomical location in dictating how biofilm influences inflammatory responses and its impact on bacterial clearance.

## Introduction

**Figure 1:**
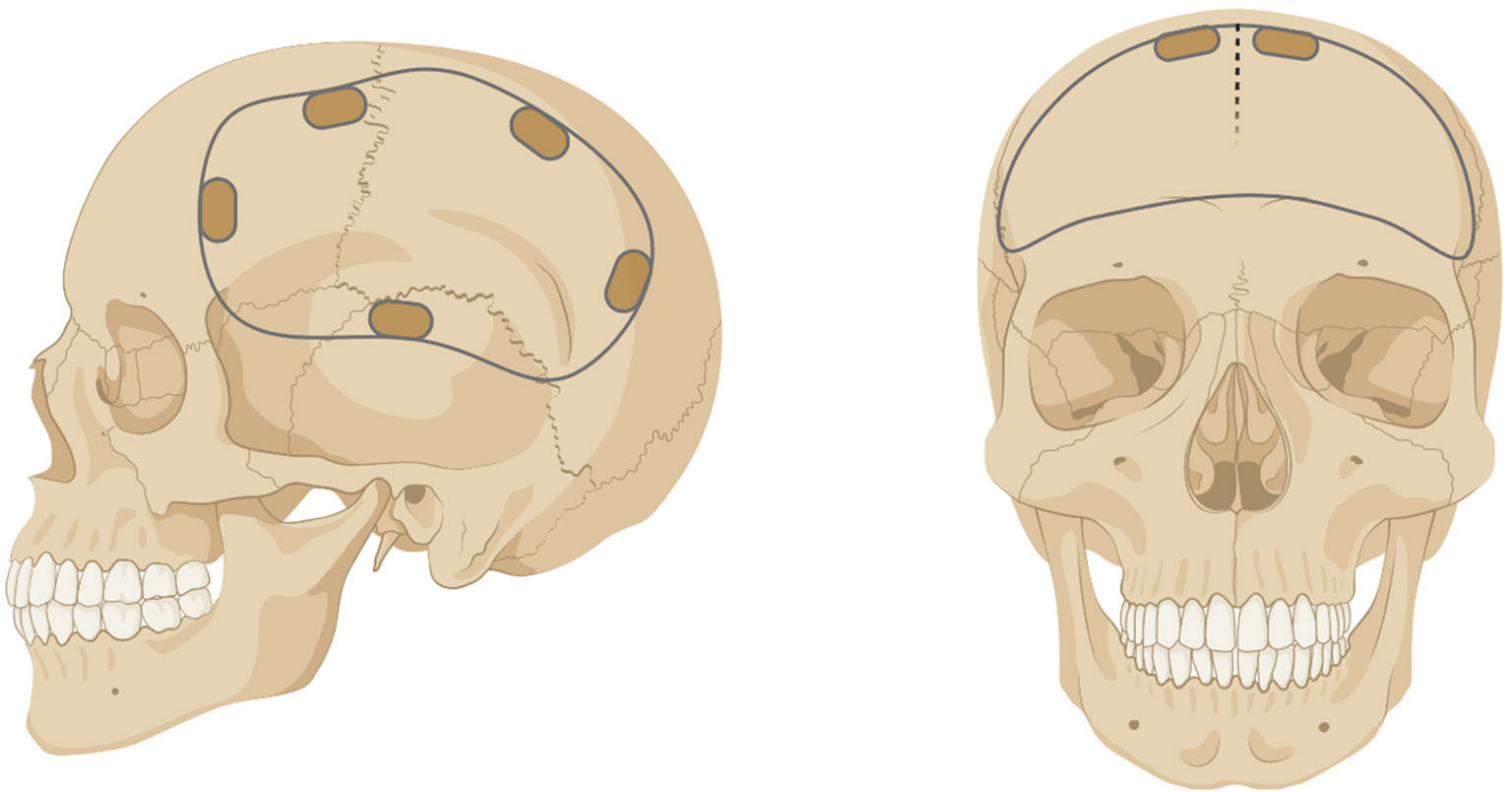
Example of a unilateral (left) or bilateral (right) craniotomy/craniectomy. Figure created with BioRender.

Craniotomy and decompressive craniectomy are neurosurgical procedures where part of the skull (i.e. bone flap) is removed to access the brain (**Figure 1**). Craniotomy involves the temporary removal of the bone flap for procedures that include tumor resection, localization and resection of epileptogenic foci, and aneurysm clipping, where the bone is replaced intraoperatively (1). Decompressive craniectomy refers to the excision of the bone flap for an extended period following traumatic brain injury, ischemic stroke, or intracranial hemorrhage to treat intracranial hypertension (2). Upon removal, the bone flap is typically cryopreserved or implanted subcutaneously in the abdomen of the patient to preserve vascularization and replaced after cerebral edema has resolved (3, 4). However, complications can occur with prolonged absence of the bone flap including extracranial herniation, trephine syndrome, hydrocephalus, seizures, and neurological dysfunction (5, 6).

Despite peri- and post-operative prophylaxis, infectious complications occur in approximately 1% to 3% of craniotomy and craniectomy procedures (7, 8). These infections are associated with a high mortality rate and poor prognosis if not treated early (9–11). In terms of therapy, the decision of whether to salvage or discard the bone flap is left to the neurosurgeon and is often dictated by the length of time from the initial surgery to the presentation of clinical signs of infection. The first option is to salvage the infected bone flap with a combination of debridement and long-term antibiotic therapy. Alternatively, the bone flap can be discarded and, following extended antibiotic treatment, a cranioplasty is performed to correct the acquired skull defect with an autologous bone graft or prosthesis (12–15). Prior craniotomy infection increases the risk for re-infection, which may result from the outgrowth of residual bacteria that were not eliminated due to biofilm formation (see below).

Approximately one half of craniotomy/craniectomy infections are attributed to *S. aureus* (7, 16–18), a gram-positive pathogen that forms a biofilm on native bone (19). Infections can be caused by other bacteria and fungi, although these occur at a much lower rate (8, 17). Around 30% of the human population is colonized with *S. aureus*, typically in the nares and skin, and colonized individuals have an increased risk for invasive *S. aureus* infection (20). Although pre-surgical screening for *S. aureus* carrier status is routinely performed to decolonize carriers prior to orthopedic surgery (21), this approach has not been universally adopted in neurosurgery for patients that require a craniotomy/craniectomy.

*S. aureus* is a versatile pathogen, which is attributable to several features. First, the organism is prone to genetic adaptation, particularly the ability to acquire antibiotic resistance. An example is the *mecA* cassette that affords *S. aureus* resistance to the entire class of methicillin antibiotics (22). Second, *S. aureus* expresses an extensive repertoire of virulence factors that promote its pathogenesis and interfere with host immune recognition and bacterial clearance. These include cell surface attachment factors, capsular polysaccharides, enzymes, pore-forming toxins, superantigens, and numerous immune modulatory molecules (23–28). In addition to encoding a myriad of virulence factors, *S. aureus* can form biofilm that represents another virulence determinant (29). Biofilms are complex microbial communities surrounded by a matrix composed of extracellular DNA (eDNA), protein, and polysaccharide (29). The organization of bacteria within a biofilm creates microdomains with differential access to oxygen and nutrients leading to a sub-population of organisms that are less metabolically active, referred to as persisters (30, 31). Because most antibiotics target bacterial cell wall and protein synthesis, the metabolic dormancy of some biofilm-associated bacteria is responsible for the well-known antibiotic tolerance of biofilm. Compared to planktonic bacteria, the mechanisms responsible for *S. aureus* biofilm to evade immune-mediated clearance are only beginning to be understood. Work from our group and others has shown that *S. aureus* biofilm evades Toll-like receptor (TLR)-mediated recognition (32, 33), inhibits phagocytosis (33–37), and induces the recruitment of granulocytic-myeloid-derived suppressor cells (G-MDSCs) that inhibit monocyte/macrophage proinflammatory activity (38–40). Recent work has demonstrated that *S. aureus* metabolites (D- and L-lactate) play a key role in inducing epigenetic changes in G-MDSCs and macrophages to promote the production of the anti-inflammatory cytokine IL-10 and biofilm persistence (41).

Novel therapeutic approaches for *S. aureus* infection continued to be explored, since most antibiotics have poor efficacy against biofilm and a fine balance must be achieved with currently available antibiotics to reach an optimal minimum inhibitory concentration (MIC) during chronic administration while minimizing toxicity (31, 42). An effective vaccine against *S. aureus* has remained elusive (43, 44). This is likely explained by the fact that the organism can cause a wide range of infections with distinct attributes, and that it expresses numerous virulence factors that impair host immunity. The latter point has recently been shown to play an important role in a mouse model of *S. aureus* bacteremia where immunization with *S. aureus* toxoids reduced mortality, bacterial burden, and organ dysfunction (45). A better understanding of *S. aureus* colonization dynamics, how the organism interacts with different leukocyte populations, and influences of the local tissue milieu will be necessary to develop improved therapeutics for infections caused by *S. aureus*.

## Mouse *S. aureus* Craniotomy Model: Similarities to Human Infection

As mentioned above, *S. aureus* is a major cause of infectious complications following craniotomy (7, 9, 10); therefore, our laboratory developed a mouse model of *S. aureus* craniotomy infection to understand the immune mechanisms responsible for bacterial persistence (19). In the mouse model, a craniotomy is performed and the bone flap is colonized with *S. aureus*, which leads to biofilm formation on the bone and chronic infection in both the brain and subcutaneous galea that cannot be cleared with systemic antibiotics (46). Importantly, the mouse model shares several features with human craniotomy infection. This includes a conserved biofilm structure on the bone flap as revealed by scanning electron microscopy with similarities in extracellular matrix deposition, foci of bacterial aggregates on the bone flap, and complex tower-like structures (19). In addition, magnetic resonance imaging (MRI) revealed galeal inflammation with superficial cortical brain involvement (19), which is also an attribute of human infection and supports the translational relevance of the mouse model.

## Compartmentalization of Immune Responses During *S. aureus* Craniotomy Infection

The CNS was once considered immune privileged based on the restrictive attributes of the blood brain barrier (BBB) (47–49). However, it is now clear that immune responses do occur in the CNS in a wide range of neurodegenerative and infectious diseases, and immune surveillance of the CNS takes place in the absence of pathology (50–52). Over the past decade, our laboratory has characterized the immune responses to *S. aureus* biofilm infection in both the CNS (craniotomy-associated infection) and periphery (prosthetic joint infection (PJI)) (19, 40, 41, 46, 53–56). Comparisons between these models clearly show that the immune responses elicited are distinct, which will be discussed later in this review, reflecting influences of the local tissue milieu. The impact of infection site and how this shapes the subsequent immune response has also been reported by other groups and emphasizes the need to understand niche-specific factors that influence *S. aureus*-immune crosstalk (57–61).

**Figure 2:**
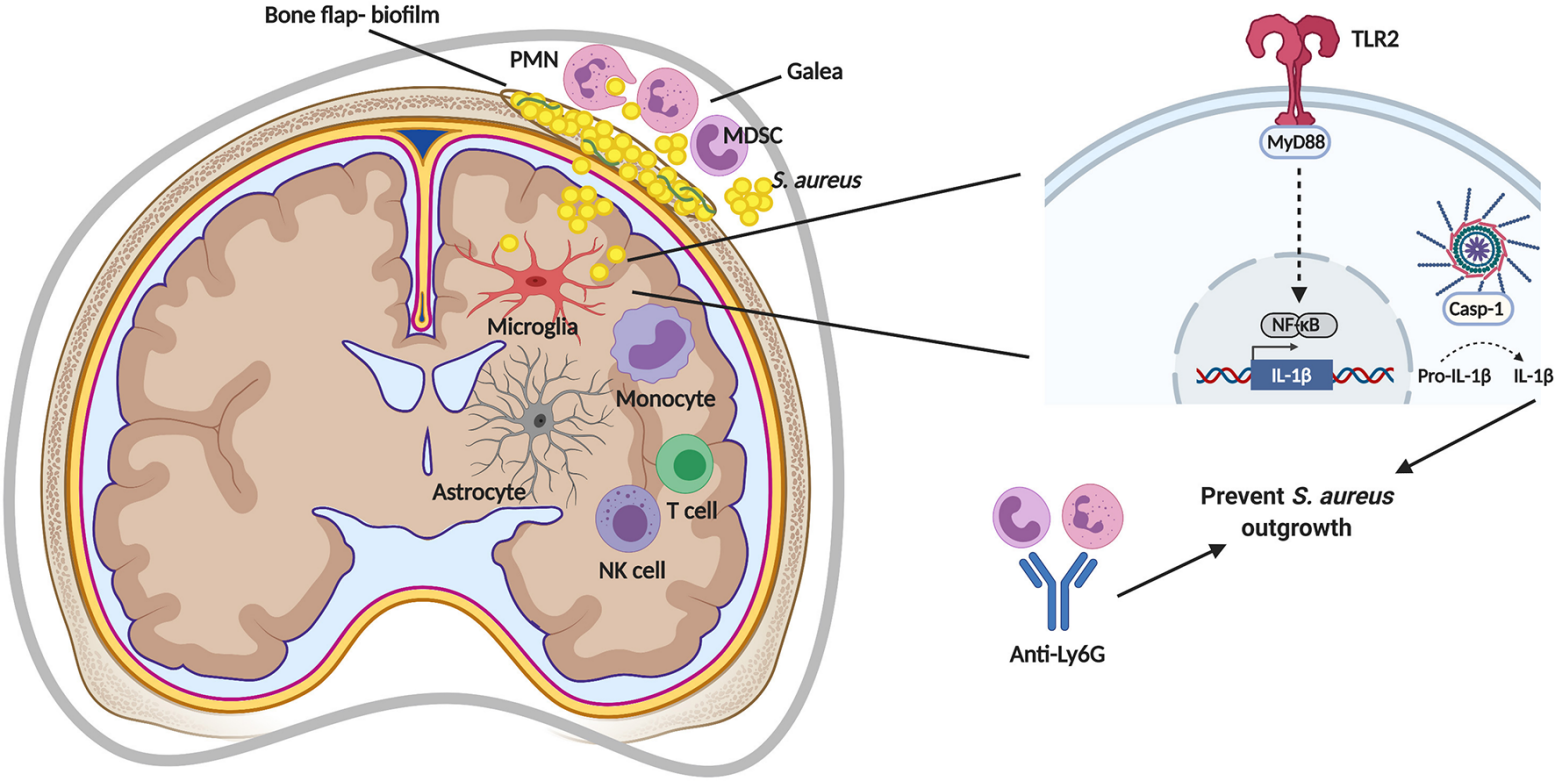
Immune responses during *S. aureus* craniotomy infection. *S. aureus* biofilm formation of the bone flap elicits a unique inflammatory response in the subcutaneous galea and brain. TLR2-mediated signaling *via* MyD88 induces pro-IL-1β production that is cleaved by a caspase-1 (casp-1)-containing inflammasome for secretion and to prevent *S. aureus* outgrowth. *S. aureus* containment is also mediated by neutrophils (PMNs), as shown by depletion using anti-Ly6G. MDSC, myeloid-derived suppressor cell; NF-κB, nuclear factor-kappa B; NK, natural killer. Figure created with BioRender.

Even within a given infection, compartmentalization of immune responses can be observed. An example is the *S. aureus* craniotomy model where patterns of leukocyte recruitment and inflammatory mediator expression are distinct in the brain vs. subcutaneous galea despite both tissues being exposed to bacteria on the bone flap. For example, monocytes, innate lymphoid cells (NK and ɣδ T cells), and T cells are preferentially recruited to the brain, whereas G-MDSCs and neutrophils (PMNs) are the main leukocyte infiltrates in the galea (**Figure 2**) (19, 56, 62). The attributes of these cell types and their role during *S. aureus* infection will be described in more detail below. Likewise, the expression of chemokines, such as CCL2 (monocyte chemoattractant protein-1; MCP-1) and CXCL10 (interferon-inducible protein 10 kDa; IP-10) are higher in the brain (19, 56), which coincides with the enhanced recruitment of monocytes and lymphocyte populations. In the galea, chemokines responsible for PMN and G-MDSCs influx (CXCL2; macrophage inflammatory protein-2; MIP-2) are generally enriched (19, 56) in agreement with the preferential recruitment of these populations to this compartment.

Interactions between the immune system and CNS are not only important for controlling infection, but also for maintaining homeostatic functions including neurogenesis, behavior, and neuronal activity (63–66). Therefore, a delicate balance must be achieved to elicit sufficient inflammatory responses to clear infection without becoming overactive, which can lead to collateral tissue damage. Indeed, many bacterial infections in the CNS, including those caused by *S. aureus*, result in areas of tissue necrosis that vary according to infection severity. CNS biofilm infection represents an interesting dilemma since the chronicity of these infections is not characteristic of an overactive immune response, but instead one that is non-productive or anti-inflammatory. In this instance, CNS pathology may be mediated by products released from the biofilm, such as bacterial proteases, nucleases, or *via* the consumption of metabolites that are critical for CNS function (i.e. glucose). In the following section, we present an overview of the different immune populations associated with *S. aureus* craniotomy infection and their functional attributes.

## Microglia and Myeloid Cells Associated With *S. aureus* Craniotomy Infection

### Microglia

Microglia comprise approximately 10% of brain parenchymal cells, which undergo slow proliferation throughout the lifespan of an organism to maintain their numbers (67, 68). Historically, there was much debate about the origin of microglia, where earlier dogma considered microglia to be bone marrow-derived; however, this has now been definitively disproved (69, 70). It is now well established that microglia arise from erythromyeloid precursors in the primitive yolk sac that migrate to the brain where they differentiate into microglia (71, 72). Microglia continuously survey the CNS parenchyma by expanding and retracting their processes to monitor the extracellular milieu, surrounding neurons and other glial cells, as well as to detect invading pathogens and CNS damage (73–75). Microglia play a key role in the phagocytosis of microbes, apoptotic cells, and protein aggregates, and produce a wide array of inflammatory mediators based on their diverse repertoire of pattern-recognition receptor (PRR) expression. Pertinent to *S. aureus*, microglia express 1) TLR2 and TLR9 that recognize bacterial lipoproteins and non-methylated CpG DNA motifs, respectively; 2) nucleotide-binding oligomerization domain-containing protein 2 (NOD2), an intracellular PRR that senses muramyl dipeptide, a component of peptidoglycan that is abundant in the cell wall of *S. aureus* and other gram-positive bacteria; and 3) CD14 (76–79). Following PRR activation, microglia produce a wide array of proinflammatory cytokines and chemokines (i.e. TNF-α, IL-6, IL-1β, IL-12, and CCL2) as well as reactive oxygen and nitrogen species (ROS, RNS). These mediators have pleotropic effects including promoting BBB permeability (TNF-α, IL-6, IL-1β), leukocyte recruitment (CCL2) and activation (TNF-α, IL-6, IL-1β, IL-12) and bactericidal activity (ROS/RNS), but they can also negatively impact neuronal function and survival if not tightly regulated (80). Therefore, the induction of anti-inflammatory mechanisms are critical to resolve inflammation and promote tissue repair, which are largely mediated by cytokines such as IL-10 and transforming growth factor-beta (TGF-β) (81–83). The chronicity of *S. aureus* craniotomy infection suggests a potential imbalance towards an anti-inflammatory state. This is supported by the presence of immune suppressive G-MDSCs as well as PMN and monocyte infiltrates that also possess anti-inflammatory activity as reflected by their ability to inhibit T cell activation (62).

Although as myeloid cells microglia and bone marrow-derived macrophages have distinct origins, they share many attributes including similarities in marker expression, cytokine production, and dependence on macrophage colony-stimulating factor-1 (CSF-1) for survival and proliferation (84–87). During CNS inflammatory conditions, it is not possible to discriminate between microglia and infiltrating macrophages in histological sections since activated microglia transform to an amoeboid morphology that is indistinguishable from macrophages. However, microglia can be discerned from infiltrating monocytes and macrophages by flow cytometry based on CD45 expression (macrophages are CD45^high^ whereas microglia are CD45^low/intermediate^) (88). Furthermore, advances in next generation sequencing (NGS) and scRNA-seq have identified unique transcriptional profiles of resident microglia versus infiltrating macrophages (89–92), which has led to the identification of markers that are either uniquely (Tmem119, P2YR12, Hexb) or more highly expressed (CX3CR1) in microglia compared to macrophages to aid in their discrimination. Emerging studies from our laboratory have established the transcriptional heterogeneity of resident microglia and macrophage infiltrates in the brain during *S. aureus* craniotomy infection with the goal of identifying unique markers that will enable the purification of distinct microglial/macrophage clusters to understand their functional role and whether this shapes the chronicity of CNS biofilm infection (62).

### Monocytes and Macrophages

Monocytes are bone marrow-derived and invade the inflamed CNS in response to injury or infection primarily *via* a CCR2-dependent pathway (93–95). Studies have implicated monocytes in the pathogenesis of several neurological diseases, including experimental autoimmune encephalomyelitis (EAE) an animal model of multiple sclerosis (MS), where preventing monocyte recruitment or monocyte depletion reduced disease severity (96, 97). To date, fewer studies have examined monocyte responses to *S. aureus*. TLR2 has been shown to regulate *S. aureus* intracellular survival in monocytes *via* a type I IFN pathway and induce IL-10 production to limit T cell responses (98, 99). Future studies are needed to assess the role of monocytes during craniotomy infection since they represent the predominant leukocyte infiltrate in the brain following *S. aureus* invasion (56).

Upon migrating into tissues, bone marrow-derived monocytes differentiate into macrophages. Macrophages are professional phagocytes that, along with microglia, play an important role in eliminating debris and apoptotic cells during inflammation in the brain parenchyma, which is critical for maintaining CNS homeostasis (100). There are three resident macrophage populations associated with the CNS, namely perivascular, meningeal, and choroid plexus macrophages. Perivascular and meningeal macrophages are derived from yolk sac progenitors, whereas choroid plexus macrophages originate from both yolk sac progenitors and the bone marrow (71). Each macrophage population possesses unique phenotypes with different capacities for self-renewal (71, 101), which is likely influenced by the local tissue microenvironment.

Macrophages are critical effector cells during infection, with planktonic *S. aureus* inducing robust proinflammatory cytokine and ROS/RNS production and bactericidal activity (102, 103). However, *S. aureus* expresses a number of virulence determinants to counteract macrophage effector mechanisms. This includes the production of molecules that interfere with TLR2-dependent recognition (104), such as lipase (Geh) (105), staphylococcal superantigen-like protein 3 (SSL3) (106), and molecular mimicry *via* blocking the Toll-interacting receptor (Tir) domain of TLR2 (107, 108). In addition, the paired-immunoglobulin-like receptor (PIR)-B contains an inhibitory immunoreceptor tyrosine-based inhibition motif (ITIM) that, upon binding *S. aureus* lipoteichoic acid, dampens proinflammatory cytokine production (109, 110). Biofilm formation by *S. aureus* also represents another virulence determinant to escape macrophage effector functions. *S. aureus* biofilm evades TLR2-mediated recognition, and macrophage invasion into biofilm is limited *in vitro* and *in vivo*. This biases cells towards an anti-inflammatory profile that prevents bacterial clearance (33). Macrophages are not capable of phagocytosing *S. aureus* biofilm (33), which leads to frustrated phagocytosis and cell death that is mediated, in part, through the action of toxins (α-toxin and leukocidin AB) (37). The adoptive transfer of proinflammatory macrophages in a mouse model of *S. aureus* catheter-associated infection was shown to transform the biofilm milieu into a proinflammatory state concomitant with reduced arginase-1 (Arg-1) expression, which decreased biofilm burden in a MyD88-dependent manner (34). Metabolic reprogramming of monocytes/macrophages to promote their proinflammatory activity was also capable of reducing biofilm burden in a mouse model of PJI (55) highlighting the importance of augmenting macrophage proinflammatory activity as a novel approach to target chronic biofilm infection. The impact of monocytes and macrophages in the pathogenesis of *S. aureus* craniotomy infection and how their metabolic status influences their inflammatory properties remains to be determined and represents an area of investigation in our laboratory.

### Neutrophils

PMNs are bone marrow-derived and are released into the circulation at a rate of 10^9^/kg body weight per day (111). PMNs are the first leukocytes recruited to sites of bacterial infection by several chemoattractants including IL-8 (functional mouse homologs are CXCL1 and CXCL2), complement split products (C3a and C5a), and formylated peptides released from bacteria (f-Met-Leu-Phe). Upon extravasation, PMNs exert potent bactericidal activity through the action of antimicrobial peptides and granule enzymes, ROS production, neutrophil extracellular traps (NETs), and phagocytic activity (112, 113).

*S. aureus* encodes an extensive repertoire of virulence factors to escape PMN killing. Molecules such as chemotaxis inhibitory protein of *Staphylococcus aureus* (CHIPS), formyl peptide receptor-like 1 (FPRL1), staphopain A, and staphylococcal superantigen-like proteins (SSLs) disrupt various aspects of PMN priming, activation, chemotaxis, and adhesion (23, 114, 115). Moreover, *S. aureus* secretes proteins that target complement and opsonophagocytosis (protein A), antioxidants that neutralize ROS (catalase, superoxide dismutase), and numerous toxins with pore-forming properties (phenol soluble modulins, leukocidins, α-toxin) all of which function to diminish PMN antibacterial activity (116–123). Individuals with mutations in NADPH oxidase (chronic granulomatous disease; CGD) are highly susceptible to severe and life-threatening *S. aureus* infections highlighting the critical role of PMNs in bacterial containment (124). Although PMNs are recognized for their beneficial roles during injury or infection (125), dysregulated activity has been implicated in tissue pathology originating from bystander damage *via* products released from activated PMNs (126–128).

The majority of PMNs during *S. aureus* craniotomy infection localize to the galea and bone flap, whereas PMN infiltrates are minimal in the brain. These patterns are similar to the profiles of G-MDSCs recruitment (56). Although earlier work in the *S. aureus* craniotomy infection model suggested an important role for PMNs in preventing bacterial outgrowth, this was with an anti-Gr-1 depletion strategy (19). This approach also targets Ly6C^+^ monocytes, since the Gr-1 antibody recognizes both Ly6G and Ly6C (129). The functional importance of PMNs during *S. aureus* craniotomy infection was recently demonstrated by our laboratory using a more selective targeting approach (i.e. Ly6G depletion). PMNs were critical for bacterial containment, although the chronicity of craniotomy infection indicates that PMNs are not capable of eliminating biofilm in the wild type setting (62).

### Myeloid-Derived Suppressor Cells

Under physiological conditions, immature myeloid cells undergo maturation in the bone marrow, whereupon they are released and migrate to tissues to become effector macrophages, dendritic cells, or PMNs. During pathologic conditions such as cancer, infection, or chronic inflammation, proinflammatory mediators and/or endoplasmic reticulum (ER) stress drive immature myeloid cell expansion and their conversion into MDSCs (130–132). The growth factors G-CSF and GM-CSF are important for stimulating MDSC expansion with proinflammatory cytokines (IL-1β, TNF-α, IL-6) playing a key role in their activation (133–135). MDSCs exert potent immune-regulatory activity through several mechanisms, including suppressing macrophage and dendritic cell proinflammatory activity, promoting regulatory T cell (Treg) activation, and inhibiting CD4^+^ and CD8^+^ T cells. These effects are mediated by the action of several molecules including Arg-1, nitric oxide (NO), TGF-β, IL-10, cyclooxygenase-2 (COX-2), and ROS (133, 134, 136, 137). Through these mechanisms, MDSCs limit inflammation to perpetuate chronic infection by suppressing immune effectors that are important for disease resolution (138).

MDSCs consist of two groups referred to as granulocytic (G-MDSCs or PMN-MDSCs) and monocytic (M-MDSCs) that share phenotypic characteristics with PMNs and monocytes, respectively. Each MDSC subset utilizes distinct mechanisms to attenuate immune responses, where generally M-MDSCs suppress using NO (134, 139, 140), whereas G-MDSCs utilize ROS (141, 142). MDSCs have been best characterized in cancer; however, reports describing their importance during infection and chronic inflammation have emerged in recent years (143–146). Our group has been investigating MDSC-*S. aureus* biofilm crosstalk since 2014, and the role of MDSCs during *S. aureus* infection has been confirmed by other groups (38–41, 147–150). In response to peripheral *S. aureus* biofilm (i.e. PJI), G-MDSCs are critical for inhibiting monocyte/macrophage proinflammatory activity primarily through IL-10 production (38–41). IL-10 is induced by lactate released from *S. aureus* biofilm, which inhibits HDAC11 to induce epigenetic changes at the *Il-10* promoter as well as other genes (41). G-MDSCs are also enriched in humans during PJI, and are expanded in the blood following orthopedic infection (151, 152). This suggests that they may play an important role in dictating infection persistence and/or susceptibility, respectively. MDSCs are the major leukocyte infiltrate in the galea and bone flap during *S. aureus* craniotomy infection, but are rare in the brain parenchyma (56). The transcriptional profiles of MDSCs during craniotomy infection identified them as G-MDSCs, which were shown to inhibit PMN *S. aureus* bactericidal activity (62). The effector molecules that are critical for G-MDSC suppressive activity in the context of *S. aureus* craniotomy infection remain to be identified.

## Other Cell Types in the Brain During *S. aureus* Craniotomy Infection

### Astrocytes

Astrocytes are the most abundant cell type in the CNS parenchyma. They play a key role in maintaining neuronal homeostasis, BBB integrity, and can contribute to immune responses by the production of a wide array of chemokines that promote leukocyte recruitment to the CNS (153–155). *S. aureus* triggers TLR2 signaling in astrocytes and the secretion of NO, IL-1β, and TNF-α *via* NF-kB- and MAPK-dependent pathways (156). Other studies have shown that TLR activation induces astrocyte chemokine production (CCL2, CCL3, CCL5) and augments adhesion molecule expression (157, 158). In astrocytes, the intracellular pattern recognition receptor NOD2 was shown to activate NF-kB leading to IL-6, TNF-α, and co-stimulatory molecule expression, which amplified the anti-bacterial immune response (159). Based on their ability to influence immune responses *via* robust chemokine production, it is possible that astrocytes play an important role in leukocyte recruitment to the brain during *S. aureus* craniotomy infection. Of particular interest would be the production of monocyte, NK cell, and ɣδ T cell chemokines, since these cell types represent the most abundant leukocyte infiltrates in the brain (62). Studying this will require the use of transgenic mouse models where candidate chemokines are selectively depleted in astrocytes (i.e. Aldh1l1-Cre) (160), since it is not feasible to eliminate astrocytes due to their essential role in brain physiology. However, assigning a biological role to only one chemokine in the context of craniotomy infection might prove difficult based on the known redundancy in chemokine actions (161). An alternative approach would be to identify the chemokine receptors that are required for monocyte, NK cell, and ɣδ T cell recruitment into the brain and leverage this information to identify the responsible chemokines.

### T Cells

T cells participate in CNS immune surveillance and are important for normal learning and memory, behavior, and neurogenesis through IL-4 and IFN-ɣ production (162–164). It is important to note that these effects occur in the absence of CNS pathology when T cell numbers are low, since it is well recognized that increased T cell recruitment to the brain during diseases such as MS or normal aging is associated with adverse outcomes (164–166).

Interestingly, there are conflicting reports on the role of T cells during *S. aureus* biofilm infection in the periphery. In a model of tibial infection where titanium implants were pre-coated with *S. aureus* (high infectious inoculum), a beneficial role for Th2 and Treg cells in promoting biofilm clearance has been reported (167). In contrast, in a mouse model of *S. aureus* PJI with a low infectious inoculum, few T cells were observed, and tissues from PJI patients have fewer T cell infiltrates compared to individuals with aseptic trauma (39, 151). The reasons for this discrepancy are unclear, but they are likely influenced by differences in the infectious dose, background strain of mice, or site of implant infection. T cells are observed in the brain during *S. aureus* craniotomy infection, but are largely absent from the galea and bone flap (62). This pattern of recruitment suggests that T cells may play an important role in regulating the host response to craniotomy infection in the brain, but it remains to be determined whether this contributes to infection chronicity, or if T cells are a bystander population and do not significantly influence *S. aureus* biofilm persistence.

## Immune Responses During *S. aureus* Craniotomy Infection

As discussed earlier, an intriguing aspect of *S. aureus* craniotomy infection is the generation of distinct immune responses within the CNS (brain) versus peripheral compartments (galea and bone flap; **Figure 2**). Although it might be expected that immune responses would differ in the brain compared to the periphery, prior studies in a mouse model of *S. aureus* brain abscess revealed that inflammatory changes in the brain were similar in nature to peripheral abscesses as described below. Therefore, the *S. aureus* craniotomy model can be leveraged to elucidate signals that orchestrate unique inflammatory events in the brain vs. periphery, which may lead to tailored therapies for each compartment. This would be particularly useful given the fact that CNS neurons cannot regenerate and, as such, eliciting efficient pathogen neutralization without excessive bystander damage that can accompany inflammation is paramount. During *S. aureus* craniotomy infection, PMN influx is significantly higher in the galea compared to the brain despite the presence of CXCL1 and CXCL2 in both compartments (19). This might be explained by the higher bacterial burden in the galea compared to the brain (typically 1-log), although both surfaces of the bone flap are colonized with *S. aureus* (19). Furthermore, the meninges that cover the surface of the brain are patrolled by resident meningeal macrophages that likely serve to limit *S. aureus* invasion into the brain. Interestingly, meningitis is not observed at the histological level in the *S. aureus* craniotomy infection model (19, 56), suggesting that any bacteria that detach from the ventral aspect of the bone flap are prevented from significant expansion in the subarachnoid space. The predominance of PMNs in the galea suggests that they are important for containing infection. This was supported by the finding that mice treated with a Gr-1 antibody became more moribund with increased bacterial burden (19). However, the Gr-1 antibody targets both Ly6G^+^ and Ly6C^+^ cells, meaning that not only were Ly6G^+^ PMNs (and G-MDSCs) depleted, but also Ly6C^+^ monocytes (168). A subsequent study from our group utilizing selective depletion of Ly6G^+^ cells has revealed that PMNs are critical for preventing *S. aureus* outgrowth during craniotomy infection, yet mice were not moribund unlike that observed following Gr-1 antibody treatment (62). By extension, this suggests that monocytes/macrophages also play a protective role during craniotomy infection, since they were not depleted with anti-Ly6G. An interesting observation is that although G-MDSCs are also targeted by anti-Ly6G, the removal of this suppressive population did not improve biofilm clearance. Instead, the opposite was observed, suggesting that PMNs are the main driver of biofilm containment in the craniotomy infection model (62).

Based on the chronicity of *S. aureus* craniotomy infection in the mouse model (at least 9 months, the latest time point examined to date), it might be assumed that there is minimal involvement of proinflammatory mechanisms (46). However, as alluded to above, there is some degree of proinflammatory tone during craniotomy biofilm infection because PMN/monocyte depletion with anti-Gr-1 results in rapid *S. aureus* outgrowth in the brain, galea, and on the bone flap (19). Another indication that proinflammatory cytokines are critical for bacterial containment has been through the examination of TLR signaling pathways. Our initial study examined MyD88, the adaptor molecule that facilitates signaling through all TLRs (except TLR3), IL-1R, and IL-18R, and is a critical factor in innate immune defense (169). MyD88 KO mice were extremely susceptible to *S. aureus* craniotomy infection with a significant reduction in PMN infiltrates and proinflammatory mediator production (CXCL1 and IL-1β) that resulted in increased bacterial burden in the brain, galea, and bone flap (19). These phenotypes combined with the enhanced morbidity of MyD88 KO mice were akin to the effects seen during anti-Gr-1 treatment where essentially all innate immune effectors were depleted (PMNs and monocytes) (19). The importance of TLRs and downstream effector mechanisms was further demonstrated by our recent work that revealed a crucial role for TLR2 and caspase-1 during *S. aureus* craniotomy infection, primarily *via* IL-1β action (56). Interleukin-1β is produced in an inactive pro-form that requires proteolytic cleavage by the inflammasome whose active moiety is caspase-1 (170). Inflammasome activation involves two signals; the first being delivered by a PRR, such as TLR2, which leads to the production of inflammasome subunits and pro-IL-1β. The second signal can be delivered by any number of stimuli depending on the type of NLR sensor (i.e. NLRP3, NLRC1, etc.) that results in inflammasome assembly and caspase-1 activation (170). Mice lacking either functional TLR2 or caspase-1 displayed increased bacterial burden in the brain, galea, and bone flap, which coincided with significant decreases in the production of several proinflammatory mediators including IL-1β (56). A critical role for IL-1β in bacterial containment was established by the fact that treatment of caspase-1 KO mice with IL-1β-containing microparticles returned the exaggerated bacterial burden in these animals to levels observed in WT mice (56). These findings revealed the essential role of the TLR2/caspase-1/IL-1β axis in bacterial containment during *S. aureus* craniotomy infection (**Figure 2**). The importance of TLR2 in preventing *S. aureus* outgrowth is intriguing given the number of *S. aureus* virulence factors that target this signaling pathway (28) as described earlier. One explanation is that these TLR2 evasion molecules have been described during planktonic growth, and it is unknown whether they are expressed during biofilm formation. In addition, it is clear that although TLR2-dependent pathways are capable of limiting *S. aureus* biofilm outgrowth, they are not sufficient to clear infection, since biofilm persists in the wild type setting. This was further demonstrated by the finding that exogenous IL-1β treatment was not able to reduce *S. aureus* burden in WT mice, revealing the recalcitrance of biofilm to proinflammatory signals (56). Interestingly, although TLR9 is an important sensor for staphylococcal DNA that is a major component of the *S. aureus* biofilm matrix (29), TLR9 had minimal impact on the course of craniotomy infection (56). This may result from the fact that TLR9 is an intracellular PRR that requires phagocytic uptake of bacteria or eDNA, and prior studies have demonstrated that *S. aureus* biofilm interferes with macrophage phagocytosis (33, 37). Collectively, these findings highlight the fact that anti-bacterial pathways are operative during craniotomy infection; however, this is difficult to appreciate in a wild type setting based on the chronicity of infection.

There are many unknowns regarding the pathogenesis of *S. aureus* craniotomy infection. One critical point relates to identifying the mechanisms responsible for biofilm persistence despite antibiotic treatment. Second, we know little about the contributions of brain-resident cells, such as microglia and astrocytes, which are capable of influencing immune responses. In addition, we have recently identified a prominent influx of NK and γδ T cells in the brain during *S. aureus* craniotomy infection (62) and it will be interesting to examine the functional significance of each population in future studies. Finally, it will be critical to identify *S. aureus* virulence determinants that are important for promoting biofilm persistence, and to evaluate whether unique *S. aureus* transcriptional signatures are observed in bacteria recovered from the brain, galea, or the bone flap where the physical biofilm resides. These are all topics for future investigation.

## *S. aureus* Craniotomy Infection and Brain Abscess: Similarities and Distinctions

Brain abscesses pose a challenging clinical problem, which can be associated with high mortality rates due to brain compression and neuronal death from associated edema (171). Pyogenic staphylococci and streptococci are among the most prevalent bacterial species associated with brain abscesses emanating from hematogenous spread (172). Abscess formation may also arise as a complication of neurosurgery or head trauma, and is commonly associated with *S. aureus* (8, 173). Anatomically, a brain abscess possesses a well-formed necrotic center containing bacteria and PMNs and is surrounded by a dense capsule composed of macrophages and myofibroblasts (174). Activated microglia and astrocytes are observed in the brain parenchyma surrounding the abscess margins along with extensive edema. Astrocytes have been shown to play an important role in regulating brain abscess pathology, since mice deficient for the astrocytic intermediate filament glial fibrillary acidic protein (GFAP) displayed increased bacterial burden, large lesion size, and diffuse leukocyte infiltration (175). These effects likely resulted from the inability to wall off the abscess, since GFAP is a cytoplasmic protein and its loss does not eliminate astrocytes in the brain parenchyma.

*S. aureus* craniotomy and brain abscess fundamentally differ based on their chronicity and degree of inflammation. In mouse models, *S. aureus* brain abscesses are shorter in duration typically resolving within 14–21 days (174); whereas during *S. aureus* craniotomy infection, bacteria are detectable on the infected bone flap, galea, and the brain for as long as 9 months (46) despite both models utilizing a similar infectious dose (i.e. 10^3^ cfu vs. 10^4^ cfu for craniotomy and brain abscess, respectively). Second, there are distinctions at the histological level. Craniotomy-associated infection is not typified by abscess formation in the brain parenchyma of WT animals (19, 56), which is obviously distinct from brain abscess where a solitary lesion is elicited (176, 177). Furthermore, brain abscesses are associated with significant edema (177, 178), whereas edema is not a prominent feature of craniotomy infection in the brain, although a purulent exudate forms in the galea (19, 56). Third, there are distinctions in the patterns of leukocyte recruitment. Craniotomy infections are typified by a more complex immune response owing to differential leukocyte recruitment across the brain, galea, and bone flap. The infected bone flap and galea are dominated by granulocytic infiltrates (i.e. PMNs and G-MDSCs) in regions that coincide with the highest bacterial burden (56). In contrast, the brain is typified by a monocytic infiltrate and an approximate 1-log reduction in bacteria compared to the bone flap and galea. Although brain abscesses are typically a solitary lesion, there is still some degree of specificity in leukocyte homing to particular niches. PMNs migrate primarily to the necrotic core, whereas macrophages are detected along the fibrotic abscess capsule (179).

**Figure 3:**
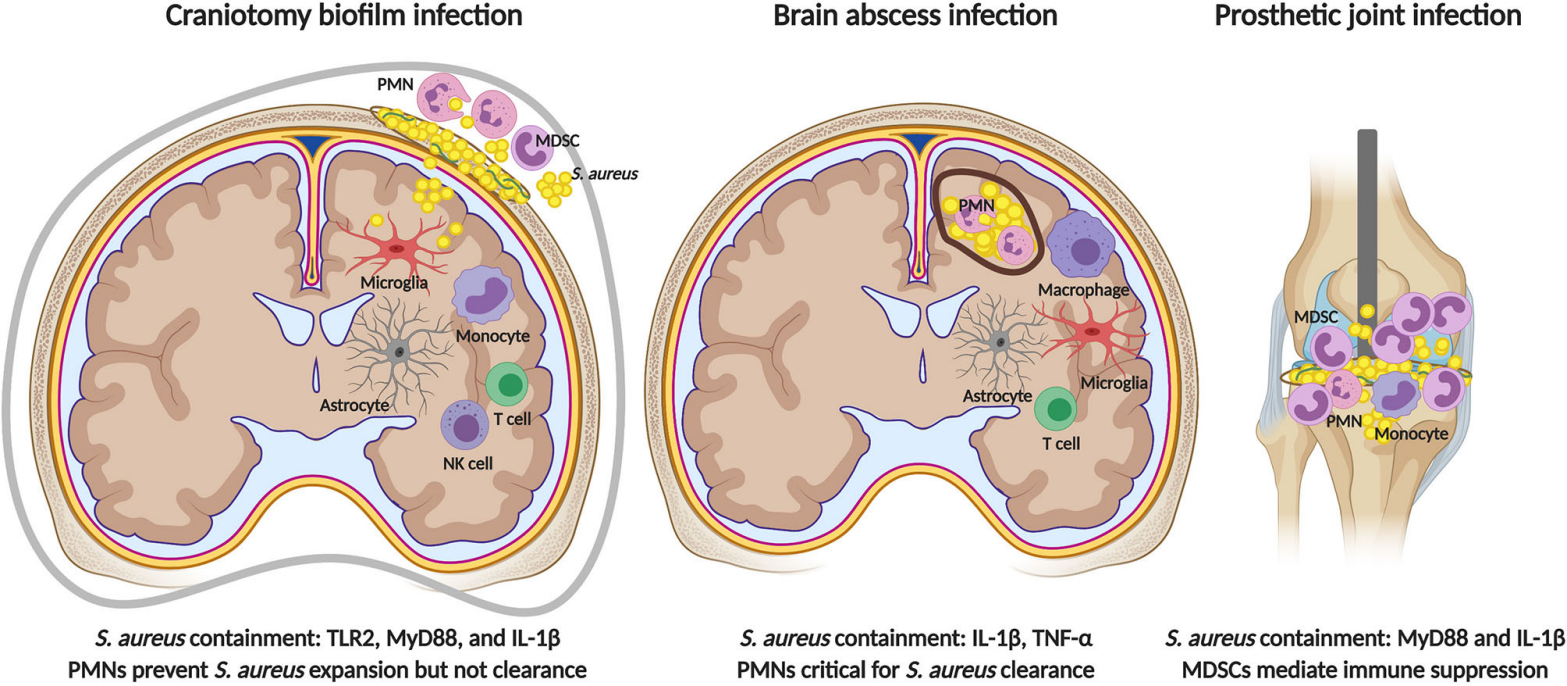
Comparisons in immune responses elicited by *S. aureus* in the CNS versus periphery. TLR2 signaling is critical for preventing bacterial outgrowth during *S. aureus* craniotomy infection (left), but is dispensable during brain abscess (center) and peripheral biofilm infection (right). MyD88-dependent signals are critical for restricting *S. aureus* growth during craniotomy and peripheral biofilm infection, and IL-1β production is key in all three models. PMNs are essential for brain abscess resolution and bacterial containment during craniotomy infection, but do not influence peripheral biofilm infection where infection chronicity is mediated by the inhibitory action of MDSCs on monocyte pro-inflammatory activity. Figure created with BioRender.

When comparing the functional roles of TLR2 and MyD88 in both models, some similarities and distinctions are noted (**Figure 3**). First, differences between *S. aureus* craniotomy infection and brain abscesses can be seen at the level of TLR2 and MyD88 involvement in bacterial containment. For example, bacterial burden was similar in brain abscesses of MyD88 KO and WT mice (178); whereas, MyD88 was critical for preventing *S. aureus* outgrowth during craniotomy-associated infection (19). A similar finding was observed with respect to TLR2 where bacterial burden was exaggerated in TLR2 KO animals during craniotomy-associated infection (56), but was comparable in brain abscesses of WT and TLR2 KO mice (180). This is particularly interesting since proinflammatory mediator production was reduced in TLR2 KO animals in both models, revealing the involvement of TLR2-independent pathways in controlling bacterial burden during brain abscess formation.

In terms of similarities between *S. aureus* craniotomy infection and brain abscess three examples are evident. First, neither model is dependent on TLR9, which could explained by the fact that TLR9 is an endosomal receptor that requires phagocytosis for pathogen-derived DNA to engage the receptor (181). *S. aureus* is known to inhibit opsonophagocytosis *via* protein A (SpA) production, and *S. aureus* biofilm attenuates macrophage phagocytic activity (33, 37, 182). Second, IL-1β plays an important role in *S. aureus* containment in both craniotomy infection and brain abscess although the pathways leading to IL-1β production differ (**Figure 3**) **(** 56, 183, 184). During *S. aureus* craniotomy infection, caspase-1 was required for maximal IL-1β production; however, there was no role for the well-characterized inflammasome protein NLRP3 or its adaptor molecule ASC (56). Therefore, the NLR sensor that is required for inflammasome assembly and caspase-1 activation during craniotomy infection remains unknown. In contrast, IL-1β release during *S. aureus* brain abscess required the AIM2 inflammasome and ASC but, similar to craniotomy infection, was NLRP3-independent (184). These findings demonstrate the involvement of distinct inflammasome platforms for triggering IL-1β release during *S. aureus* biofilm-associated craniotomy infection versus brain abscess.

Finally, *S. aureus* craniotomy and brain abscess infection share a critical role for PMNs in bacterial containment (**Figure 3**). Mice lacking CXCR2, the receptor for the PMN chemokines CXCL1 and CXCL2, showed minimal PMN recruitment into the infected brain parenchyma and higher bacterial burden in the brain abscess model (185). Likewise, anti-Gr-1 treatment mimicked these findings with exaggerated bacterial burden and a failure to limit the extent of tissue damage during brain abscess development, although monocytes were also targeted with this antibody (185). Similar phenotypes were observed during *S. aureus* craniotomy infection, where anti-Gr-1 administration led to a significant outgrowth of bacteria in the brain, galea, and bone flap within 48 h post-infection concomitant with increased morbidity (19). Subsequent studies to refine cell depletion to only the PMN/G-MDSC populations with anti-Ly6G produced similar findings with exaggerated bacterial burden (62). However, unlike anti-Gr-1 depletion where effects were observed within 48 h the anti-Ly6G phenotype was delayed in comparison, becoming significant at day 7 post-infection, and no morbidity was observed (62). Collectively, these findings reveal the essential role of PMNs in *S. aureus* brain abscess resolution and although PMNs are important for preventing bacterial outgrowth during *S. aureus* craniotomy infection, the fact that animals tolerate PMN depletion suggests the involvement of other immune populations, the identity of which remains to be determined. Furthermore, it is important to emphasize that *S. aureus* craniotomy infection persists even when PMNs are present establishing their ineffectiveness at biofilm clearance *in vivo*. This may result, in part, by the ability of G-MDSCs to inhibit PMN killing of *S. aureus* (62).

## *S. aureus* Craniotomy Versus Peripheral Biofilm Infection: Importance of Tissue Niche

*S. aureus* is a common etiologic agent of infections associated with prosthetic joints and other indwelling medical devices (186, 187). Over the years, our laboratory and others have identified numerous mechanisms used by *S. aureus* biofilm to evade host immune responses (186, 188, 189). One hallmark of *S. aureus* biofilm infection outside the CNS is the prevalence of leukocyte infiltrates that display anti-inflammatory properties. One example is MDSCs, immature myeloid cells that have the ability to suppress T cell activation and monocyte/macrophage proinflammatory activity (38). G-MDSCs are the major leukocyte infiltrate in a mouse model of *S. aureus* PJI in addition to patients with PJI (38, 40, 41, 151). Depletion of G-MDSCs and PMNs in the mouse *S. aureus* PJI model using anti-Ly6G transformed infiltrating monocytes to a proinflammatory state that led to a significant reduction in biofilm burden (38). In contrast, although G-MDSCs and PMNs are most abundant in the galea and bone flap during *S. aureus* craniotomy infection, Ly6G depletion of these cells resulted in bacterial outgrowth (62). Therefore, although both models are associated with G-MDSC infiltrates, these results suggest their differential involvement in dictating infection outcome (**Figure 3**). An alternative explanation could be differences in the abundance of PMNs in both infection models. PMN infiltrates are minimal in *S. aureus* PJI (which instead is dominated by G-MDSCs), whereas PMNs are more abundant in the galea and bone flap during *S. aureus* craniotomy infection. Therefore, the inability to contain *S. aureus* following Ly6G depletion in the craniotomy model may result from the loss of the larger PMN population that is a critical bactericidal effector.

Another distinction between PJI and craniotomy infection is the presence of innate and adaptive lymphoid populations in the latter. Both human PJI and the mouse model have few T cell infiltrates (39, 151) in agreement with the ability of the dominant G-MDSC population to inhibit T cell activation/proliferation (190). In contrast, *S. aureus* craniotomy infection is associated with significant NK and ɣδ T cell recruitment in the brain, with fewer T and B cells (62). These populations are largely absent from the galea and bone flap revealing a unique microenvironment in the brain that is responsible for the recruitment of these lymphoid populations.

A final difference between *S. aureus* biofilm infection in the CNS vs. periphery is demonstrated by the role of TLR2 in disease. As mentioned earlier, TLR2 is critical for bacterial containment during *S. aureus* craniotomy infection (56), whereas the receptor is dispensable during peripheral biofilm infection (**Figure 3**) (33). Despite the differential involvement of TLR2, the TLR/IL-1R adaptor MyD88 is plays an equally important role in preventing *S. aureus* outgrowth during both CNS and peripheral biofilm infection (19, 34, 191). This phenotype is likely driven by IL-1β given that the loss of IL-1β production or signaling results in increased bacterial outgrowth in both CNS and peripheral models of *S. aureus* biofilm infection (32, 56, 192).

## Therapeutic Strategies for Craniotomy-Associated Infection

Despite extensive precautionary measures, post-operative complications following craniotomy continue to occur with *S. aureus* responsible for approximately one-half of these infections (18). Multiple surgeries, prolonged hospital stays, and significant mortality confound the complications arising from craniotomy infections (193). Current treatment strategies include the *ex vivo* submergence of the infected bone flap in an antiseptic solution and aggressive debridement prior to re-insertion; however, these have not yet been adopted as standard-of-care practices (194, 195). Surgical drainage in combination with a prolonged antibiotic regimen can often effectively control infection (196), which is largely dictated by the interval between surgery and presentation of clinical signs of infection. In some instances, the bone flap cannot be salvaged and a cranioplasty is performed using a bone graft or alloplastic prosthesis. In either case, patients are subjected to an extended antibiotic regimen lasting for months. Because a second surgery is often required for treatment, and the potential for infection recurrence, devising novel therapeutic approaches may significantly improve the outcome of craniotomy infection without the need for more radical interventions.

Previously, our laboratory demonstrated the efficacy of administering proinflammatory macrophages to promote biofilm clearance *in vivo* (34). We leveraged this observation to evaluate the efficacy of a 3D bioprinted bone scaffold that incorporated viable macrophages and an antibiotic cocktail as a localized delivery system for the treatment of *S. aureus* craniotomy infection (46). The rationale for including viable macrophages was that they might facilitate biofilm dispersal making bacteria more susceptible to antibiotic action. The 3D bioprinted scaffold was capable of reducing established biofilm infection, since scaffold implantation at day 7 post-infection led to a significant reduction in bacterial burden and reduced BBB damage that is associated with chronic *S. aureus* infection (46). Interestingly, although macrophage incorporation into 3D scaffolds was effective at diminishing an established biofilm, this was not the case in a prophylactic paradigm; therefore, subsequent studies focused on increasing the antibiotic dose in the scaffold. This approach mitigated bacterial burden to below the limit of detection for 2 weeks; however, *S. aureus* outgrowth was observed after this period due to the loss of antibiotic from the scaffold. Therefore, second generation 3D scaffolds are currently being developed by our group that incorporate additional bioactive moieties designed to negate the outgrowth of residual bacteria after the antibiotic has exited the scaffold. Other strategies that could be leveraged to enhance therapeutic efficacy include the use of tagged nanoparticles to target a specific immune population to enhance its microbicidal activity. Furthermore, the use of systemic antibiotics once the primary biofilm burden has been reduced by 3D bioprinted scaffolds will be critical to clear residual bacteria, which was supported by our recent study (46).

Other therapeutic approaches have utilized nanoparticle-based delivery systems to augment immune cell function (197, 198). We recently employed a similar strategy to deliver IL-1β-containing microparticles to attenuate bacterial burden in caspase-1 KO mice during *S. aureus* craniotomy infection (56). It will be interesting to see how these rapidly evolving therapeutics can modulate infection in mouse biofilm models for potential translation to the clinic.

## Conclusions and Perspectives

Several studies have highlighted niche-specific differences in the composition of leukocyte infiltrates and their ensuing inflammatory responses during infection (199, 200). One example is a mouse model of visceral leishmaniasis, where parasites are cleared within 2 months following intravenous injection in the liver but are present in the spleen and bone marrow throughout the life of the animal (201). This is thought to result from alterations in adaptive immunity and macrophage function in each of these locations. Tissue-specific cues are exemplified when comparing the immune responses elicited by *S. aureus* biofilm in the periphery that, in general, are characterized by an anti-inflammatory phenotype whereas more proinflammatory responses are elicited during CNS biofilm infection. Deciphering the signals emanating from different tissues will be crucial for understanding the pathogenesis of biofilm infection and for developing selective treatment strategies to avoid adverse side effects. The use of conditional KO mice will be an extremely important tool to understand the role of immune mediators in leukocyte populations enriched in a given tissue niche. This is particularly relevant in the case of *S. aureus* craniotomy-associated infection that is more complex in terms of distinct leukocyte subsets across various tissue domains.

It is intriguing how *S. aureus* can elicit markedly different immune responses depending upon the site of infection. As discussed, *S. aureus* craniotomy infection displays a compartmentalized immune response within affected CNS regions, and recent RNA-seq studies have begun to decipher the pathways that program a given immune population in its unique niche (62). Imaging modalities such as intravital microscopy would provide an unprecedented window into *S. aureus*-leukocyte interactions and migratory patterns associated with CNS resident vs. invading immune cells in real time. Identifying the factors that modulate changes in transcriptional networks, the nature of host-pathogen interactions, and patterns of leukocyte migration would provide a better understanding of *S. aureus*-leukocyte crosstalk and ultimately pave the way for developing tailored therapeutic strategies to mitigate *S. aureus* biofilm infections within the CNS or periphery.

## Author Contributions

SD and GK contributed equally to writing the manuscript draft with clinical information provided by JM. The initial editing was performed by TK and all authors contributed to subsequent editing. All authors contributed to the article and approved the submitted version.

## Funding

The Kielian laboratory is supported by NIH grants R01 NS107369 and 3P01AI083211 (Project 4 to TK).

## Acknowledgments

The authors apologize to those whose work could not be cited due to space limitations.

## Conflict of Interest

A patent has been filed with the US Patent and Trademark Office covering the application of 3D bioprinted scaffolds for the treatment of craniotomy-associated infections that is discussed in this review (PCT/US2020/021440; TK).

The authors declare that the research was conducted in the absence of any commercial or financial relationships that could be construed as a potential conflict of interest.

## Glossary

3D: three-dimensional
Arg-1: arginase-1
AIM2: absent in melanoma 2
ASC: apoptosis-associated speck-like protein containing a carboxy-terminal CARD
BBB: blood-brain barrier
CCL2: monocyte chemoattractant protein-1
CNS: central nervous system
CSF-1: macrophage colony stimulating factor-1
CXCL1: keratinocyte chemoattractant
CXCL2: macrophage inflammatory protein-2
CXCL10: interferon-inducible protein 10 kDa
CXCR2: C-X-C receptor 2
EAE: experimental autoimmune encephalomyelitis
eDNA: extracellular DNA
ER: endoplasmic reticulum
G-CSF: granulocyte colony-stimulating factor
GFAP: glial fibrillary acidic protein
G-MDSC: granulocyte myeloid-derived suppressor cell
γδ T cell: gamma-delta
GM-CSF: granulocyte-macrophage colony-stimulating factor
IFN: interferon
IL: interleukin
KO: knockout
MAPK: mitogen-activated protein kinase
MDSC: myeloid-derived suppressor cell
M-MDSC: monocyte myeloid-derived suppressor cell
MyD88: myeloid differentiation factor 88
NET: neutrophil extracellular trap
NGS: next generation sequencing
NF-κB: nuclear factor-kappa B
NK cell: natural killer cell
NLR: NOD-like receptor
NO: nitric oxide
NOD2: nucleotide-binding oligomerization domain-containing protein 2
PJI: prosthetic joint infection
PMN: neutrophil
PRR: pattern recognition receptor
RNI: reactive nitrogen intermediate
ROS: reactive oxygen species
S. aureus: Staphylococcus aureus
scRNA-seq: single cell RNA-sequencing
Spa: protein A
TGF-β: transforming growth factor-beta
TLR: Toll-like receptor
TNF-α: tumor necrosis factor-alpha
WT: wild type
